# Neferine improves oxidative stress and apoptosis in benign prostate hyperplasia via Nrf2-ARE pathway.

**DOI:** 10.1080/13510002.2021.1871814

**Published:** 2021-01-08

**Authors:** Nabila Jahan, Apu Chowdhury, Ting Li, Ke Xu, Fen Wei, Sicen Wang

**Affiliations:** a School of Pharmacy, Xi’an Jiaotong University, Xi’an, People’s Republic of China; b Faculty of materials and chemical engineering, Yibin University, Yibin, People’s Republic of China

**Keywords:** Neferine, BPH, oxidative stress, Nrf2, apoptosis, Keap1, Bax, Bcl-2

## Abstract

**Background:**

Progression of Benign Prostate hyperplasia (BPH) is vulnerable to oxidative stress (OS) and prostatic enlargement among the aging males through apoptosis deregulation. Our present study aimed to investigate the effect of neferine (NF) in the regulation of oxidative stress and apoptosis in human BPH-1 cells.

**Methods:**

BPH epithelial cell line BPH-1 was treated with NF for 24 and 48 h. To measure oxidative stress (OS) we investigated MDA, SOD, and GST expression along with Nrf2 and its downstream gene and protein expression. Cell proliferation and apoptosis regulation was assayed with respective methods.

**Results:**

Investigation revealed NF remarkably activate Nrf2 and its downstream proteins HO-1 and NQO1 at 48 h more substantially. Nrf2/Keap1 relative gene and protein expression indicated that NF might trigger Nrf2 upregulation by decreasing Keap1 expression. Both NF concentrations (3 µM and 9 µM) were able to deplete ROS and lipid peroxidation, concurrently, up-regulated SOD and GST. NF reduced cell proliferation significantly along with the regulation of apoptotic proteins Bax, Bcl2, Cyt-C, Caspase 9, and Caspase 3 at the same time (48 h).

**Conclusion:**

This study is the first to manifest that NF may potentially regulate BPH by counterbalancing between OS and apoptosis through the activation of Nrf2-ARE pathway.

## Background

Benign prostate hyperplasia (BPH) is one of the most prevalent diseases in aging males with lower urinary tract symptoms. BPH was identified as a disease after the nineteenth century and has distinctive proliferation features through the prostate transitional zone of stromal and epithelial cells. The pervasiveness of BPH appears to be intensified after the age of 40 [1]. Merging the BPH commonness analysis, the proportion has shown to be cumulative with a prevalence of 8%−60% in older men [2]. Oxidative stress (OS) and OS mediated DNA damage is typical in adult male, which might be crucial pathogenesis for male genital tract disorder, including BPH [3,4]. OS usually neutralized by the superoxide dismutase (SOD) enzyme, and glutathione-s-transferase (GST) enzyme, but they are found relatively low in the case of BPH [5] therefore, prostate cells become vulnerable to OS. Nuclear factor erythroid 2-related factor-2 (Nrf2) is a master antioxidant inactivated with Kelch-like ECH-associated protein-1 (Keap1) complex in homeostasis condition. On the addition of an Nrf2 activator or OS response, it cleaves from Keap1 and translocate to the nucleus and binds with the antioxidant response element (ARE) gene to transcript heme oxygenase-1 (HO-1), NAD(P)H Quinone Dehydrogenase-1 (NQO1), etc. to counterbalance OS [6,7]. Nrf2 expression was found low in BPH than prostate cancer patients [8] as well HO-1 also impaired in BPH affected men [9]. NQO1 plays a critical role in maintaining redox balance between intercellular ROS and endogenous oxidative stress stimuli [10], which expression is found suppressed in prostate tumors [11].

Prostate hyperplasia is associated with apoptosis and proliferation of prostate cells [12]. Growth factors and steroid hormone interconnection regulate cell proliferation and cell death in the prostate, which become an imbalance over age in males and reduce apoptosis [13]. When DNA damage occurs due to environmental response, OS, etc., Bcl-2 associated X protein (Bax) is recruited by p53, creating pores in the mitochondrial membrane to facilitate cytochrome-C (Cyt-C) to appear into the cytoplasm. Cyt-C activates caspase-9, which further triggers caspase-3 to initiate apoptosis. B cell lymphoma-2 (Bcl-2) performs as an anti-apoptotic protein to counterbalance this programmed cell death, which counters balance Bax activation [14]. Bcl-2 is found high in BPH, where the intensity of Bax is observed low, which consequently decreases apoptosis [15] and causes prostate hyperplasia.

Neferine is a natural bisbenzylisoquinoline alkaloid obtained from the seeds of *Nelumbonucifera* [16]. Neferine has been reported as antioxidant [17], anti-tumor [18], anticancer [19], antidepressant [20], autophagy inducer [21], anti-inflammation [22] and, anti-diabetic [23]. The study shows that NF is an activator of nuclear factor erythroid 2-related factor 2 (Nrf2) activator [24] which can reduce oxidative stress. Sulforaphane, a naturally occurring isothiocyanate produced by cruciferous vegetables such as broccoli, is a potent Nrf2 activator and potentially used as anti-cancer therapy [25,26]. Sulforaphane is also reported to treat BPH condition by modulation of apoptosis and cell cycle arrest [27].

Ablative surgery, α Blockers, 5-α Reductase inhibitors (5-ARIs), and a combination of α blockers and 5-ARIs have been inaugurated to treat BPH condition [28] but also presents the risk of ejaculatory dysfunction, hypotension, tachycardia, decreased semen count [29]. So, at present effective treatable drug is still unattained. So far, there was no report of NF intervention effects on benign prostatic hyperplasia. Considering this, we investigated the antioxidant effect of NF and the regulation of the underlying apoptotic pathway in BPH-1 cells.

## Methods

### Reagents

Neferine (purity 98% by HPLC, MW 624.77) was obtained from Yuanye Bio-Tech, Shanghai, China. Sulforaphane (purity 98%) was purchased from Aladdin Industrial Corporation, Shanghai, China.

### Antibodies

GAPDH (cat#: 10494-1-AP) antibody was purchased from Proteintech (San Ying Biotechnology, Wuhan, China). Nrf2 (cat#:12721S), Keap1 (cat#:8047S), NQO1 (cat#: 62262S), HO-1 (cat#: 5853S), cleaved Caspase 3 (cat#: 9579) cleaved Caspase 9 (cat#: 9505) Cytochrome c (cat#: 11940), Bax (cat#: 5023) and Bcl2 (cat#: 3498) antibodies were purchased from Cell Signaling Technology (Danvers, MA, USA).

### Cell line and drug preparation

Human benign prostate hyperplasia epithelial cell line (BPH-1) was purchased from Leibniz Institute DSMZ-German Collection of Microorganisms & cell culture. Human BPH-1 cells were cultured in RPMI-1640 glutamax (1X) (Gibco; Thermo Fisher Scientific, Inc.), supplemented with 10% FBS, 100 IU/mL penicillin, and 100 µg/mL streptomycin (Gibco; Thermo Fisher Scientific, Inc.) and placed in a humidified atmosphere of 95% air and 5% CO2 at 37˚C with SLN, testosterone at ∼50-70% confluence. Cells were harvested 24 h & 48 h after treated with NF and SFN. Vehicle (DMSO) concentration maintained less than 0.1% with each experiment.

### Cytotoxic screening

Cell viability numbers were tested through CCK8 (YEASEN, Shanghai Yesheng Biotechnology. Co. Ltd.) to examine the cytotoxicity. Adherence cells were seeded at a density of 1 ×10^6^ cell per milliliter while pretreated with or without SFN & NF for 24 h, 48 h at diverse concentrations in the 96-well plates. NF concentrations were used 0.625 µM, 1.25 µM, 2.5 µM, 5 µM, 10 µM, 20 µM, and 40 µM. SFN concentrations were applied 3 µM, 7 µM, 15 µM, 30 µM, and 60 µM respectively. 10 µL of the CCK8 solution was added into each well, then cells were incubated at 37°C for 2 h, and the absorbance of each culture with a microplate reader (Epoch Biotech Winooski, VT, USA) at a wavelength of 450 nm.

### Quantitative real-time reverse transcription-polymerase chain reaction (qRT-PCR)

For the quantification of target gene total RNA was isolated from human prostate epithelial cell BPH-1 using Trizol reagent (cat#: 9109; TAKARA BIO INC, Japan) and reverse transcription was performed using cDNA with the PrimeScript^TM^ RT Reagent Kit (cat#: RR047Q; TAKARA BIO INC, Shiga, Japan). Oligonucleotides were designed using Primer3 software (available at http://frodo.wi.mit.edu/primer3/). The sequences of the oligonucleotide primers available publicly are shown in Table 1. qPCR amplification was performed using SYBR® Premix Ex TaqTMII (cat#: RR8208A, TAKARA BIO INC). Cycling conditions were 95°C for 10 min, followed by 40 cycles of 95°C for 15 s and 60°C for 1 min.

**Table 1.** Sequences of primers used in qRT-PCR.

Gene	Direction	Sequence
NFE2L2	Forward	5’ -TGGGCCCATTGATGTTTCTG - 3'
	Reverse	5’ -TGCCACACTGGGACTTGTGTTTA - 3'
KEAP-1	Forward	5’ -CATCGGCATCGCCAACTTC- 3'
	Reverse	5’ -ACCAGTTGGCAGTGGGACAG- 3'
HO-1	Forward	5’ -TTGCCAGTGCCACCAAGTTC - 3'
	Reverse	5’ -TCAGCAGCTCCTGCAACTCC - 3'
NQO-1	Forward	5’ -GGATTGGACCGAGCTGGAA - 3'
	Reverse	5’ -GAAACACCCAGCCGTCAGCTA - 3'
GAPDH	Forward	5’ -GTCGGAGTCAACGGATTTGG- 3'
	Reverse	5’ -TGACGGTGCCATGGAATTTG- 3'

### Western blot

Cells were cultured in big dishes until it reached 60–70%, treated with or without NF & SFN for 24 h & 48 h, respectively. Cells were placed on ice for at least 30 min and centrifuged at 12,000 × g for 10 min; afterward, the supernatant was collected and analyzed. Extracted protein concentrations were determined through the BCA Protein Assay Kit (Heart biology technology Ltd. Xian, China). Proteins were separated by 5-12% SDS-PAGE gel electrophoresis and transferred onto PVDF membranes (Merck KGaA, Darmstadt, Germany). The immune-blots were incubated with blocking (5% non-fat milk) solution for 1 h, incubated overnight at 4°C with primary antibodies (1:1000). Then secondary antibodies were diluted at 1:500–1:10,000 followed by the incubation for 60 min at room temperature, and blots were developed by Minichemi® Sagecreation (Baltimore Ave USA). Image J software (National Institutes of Health, Bethesda, MD, USA) was used to analyze and quantify signal intensities. The signals were normalized internal control by GAPDH.

### Measuring of ROS generation

Reactive oxygen species (ROS) were determined through DCFH-DA (2′,7- dichloro-fluorescein diacetate, Yuanye Biotechnology, China). Followed by the manufacturer’s guidelines, cells were incubated with DCFH-DA for 30 min in the dark at room temperature, and fluorescence intensity was detected under an inverted microscope (Nikon Eclipse Ts2). Fluorescence intensity was proportional to intracellular ROS levels. Image intensity was analyzed by Image J software (National Institutes of Health Bethesda, MD, USA).

### Flow cytometry

Flow cytometric assessment of the apoptotic rate of BPH-1 cells was determined using an Annexin V-fluorescein isothiocyanate (FITC) apoptosis detection kit (KeyGen Biotech, Nanjing, China). The cells were harvested on ice then rinsed twice in cold phosphate-buffered saline (PBS), stained at a density of 2.5×105 cells with Annexin-V FITC/PI, and investigated with a FACS Calibur Flow Cytometer (Novocyte Bioscience, San Diego, USA).

### Malondialdehyde (MDA), SOD and GST determination

BPH-1 cells were plated in 6-well plates (1 × 106 cells/well), followed with incubation for 48 h, the cells were treated with or without various concentrations of NF and SFN, then the cells were washed three times with ice-cold PBS and centrifuged at 1000 rpm for 5 min. The activities of SOD, GST were measured as the manufacturer instructions with their corresponding kits (Genmed Scientifics inc. USA). MDA concentration (Abcam USA. Cat# 118970) was also determined according to the protocol provided by the manufacturer. A microplate reader (Thermo Fisher Scientific, USA) was used to observe the readings.

### Histochemical analysis

BPH-1 cells are seeded in glass-bottom 3-well plates (Thermo Fisher Scientific, USA) were treated with or without various concentrations of NF and SFN and cell-cultured up to 60% confluence with the control group. Cell fixation obtained with 4% paraformaldehyde at room temperature for 15 min. Subsequently, the cells are incubated in a 4′, 6-diamidino-2-phenylindole (DAPI) solution for 3 min, and the images were acquired using a Laser Scanning Confocal Microscope (Nikon Eclipse Ts2, Japan).

## Results

### Effect of NF and SFN on the viability of BPH-1 cells:

**Figure 1.**
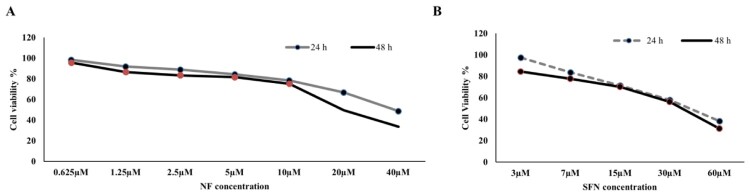
**Cytotoxic screening for NF and SFN: A.** Human BPH-1 cells were seeded at a density of 1 ×10^6^ cell per milliliter and treated with or without NF (0.625 µM, 1.25 µM, 2.5 µM, 5 µM, 10 µM, 20 µM, and 40 µM) for 24 and 48 h. Cell Counting Kit 8 was used to measure the percentage of cell viability. **B.** Human BPH-1 cell were seeded at a density of 1 ×10^6^ cells per milliliter and treated with or without SFN (3 µM, 7 µM, 15 µM, 30 µM, and 60 µM) for 24 and 48 h. (n = 5).

Concentration-dependent cell viability assay was performed for NF and SFN on the human BPH-1 cell line for 24 and 48 h. Figure 1(A), NF was introduced from 0.625 µM to 40 µM for 24 and 48 h, whereas; cell viability was reached minimum (< 40%) at 40 µM in case of both timelines. NF ranges from 2.5 µM to 10 µM showed non-toxic cell viability (≈80-70%) in both periods. So, NF 3 µM and 9 µM were selected for further experiments. The same idea was imposed on Figure 1(B) though SFN 15 µM showed safe dose (≈70%) in the case of 24 and 48 h among 3 µM to 60 µM concentration. SFN 15 µM was selected for the future experiment as positive control.

### Nrf2 regulation by NF through the Keap1/Nrf2/ARE pathway:

**Figure 2.**
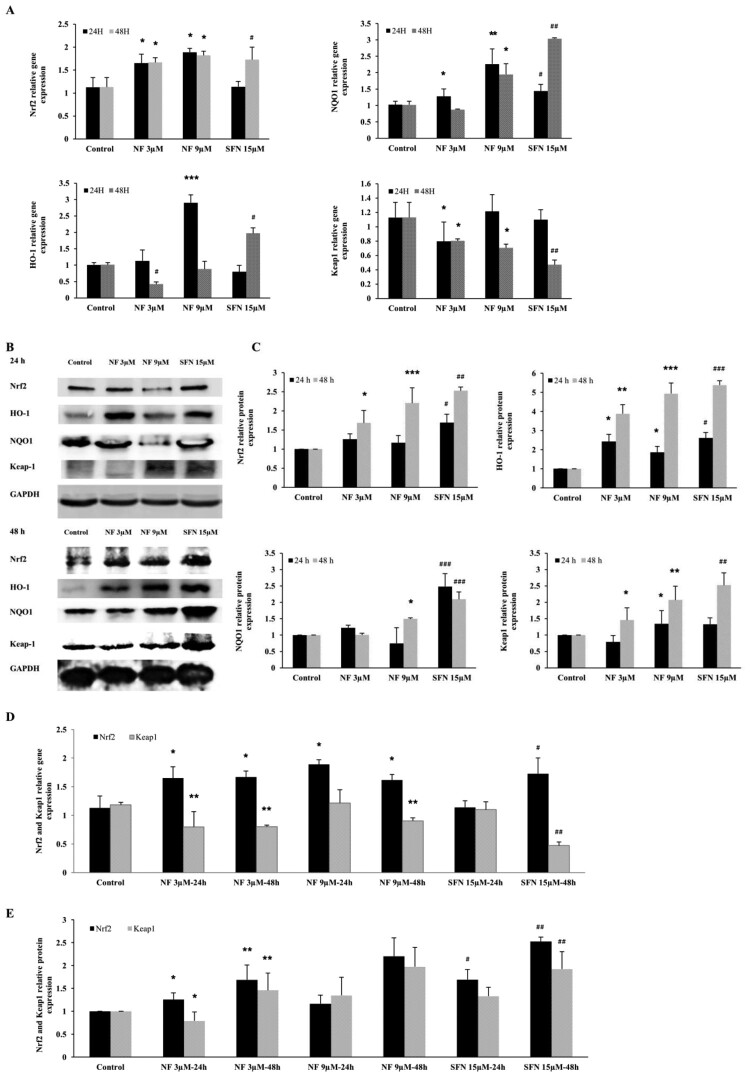
**Mechanism of Nrf2 activation by NF**: Human BPH-1 cells were treated with NF 3 µM, 9 µM, and SFN 15 µM for 24 and 48 h. **A.** The gene expression of Nrf2, HO-1, NQO1, Keap1 were normalized with GAPDH **B.** Protein expression of Nrf2, HO-1, NQO1, Keap1, GAPDH were determined with western blot. **C.** Relative protein expression of Nrf2, HO-1, NQO1, and Keap1 are normalized with GAPDH. **D.** Comparison of the Nrf2 and Keap1 relative gene expression at 24 and 48 h and all the values are normalized with GAPDH. **E.** Comparison of the Nrf2 and Keap1 relative protein expression at 24 and 48 h and all the values are normalized with GAPDH. Values are presented as means ± SD (n = 3). For NF: *P< 0.05, **P< 0.01, ***P< 0.001 compared to control group. For SFN: #*P*< 0.05, ## *P*< 0.01, ###*P*< 0.001 compared to control group (no treatment).

Regulation of critical antioxidant molecule Nrf2 and its downstream was illustrated in this current study. Nrf2, HO-1, NQO1 along Nrf2 inhibitor Keap1 were subjected to investigate with NF 3 µM, 9 µM and SFN 15 µM for 24 and 48 h. In Figure 2(A), NF 3 µM, 9 µM were able to express Nrf2 gene almost ≈1.5 fold than control in both 24 and 48 h where SFN 15 µM exhibited almost the same expression in 48 h. HO-1, NQO1, and Nrf2 deactivator Keap1 gene expression were found consequential at 24 h by NF, but SFN 15 µM was followed at 48 h. According to Figure 2 (B, C) Nrf2, HO-1, NQO1, and Keap1 protein expression were elevated at 24 and 48 h, but 48 h expression showed more noticeable where NF 9 µM demonstrated utmost expression. Figure 2 (D, E) was presented as the ratio between Nrf2 and Keap1 gene and protein expression in 24 and 48 h, although Nrf2 and Keap1 expression were inversely proportional to each other, which may explain the mode of Nrf2 activation by NF. Due to the maximum activation of Nrf2 was found in 48 h, further experiments were carried out at 48 h.

### Protection against ROS and the regulation of SOD, GST and MDA:

**Figure 3.**
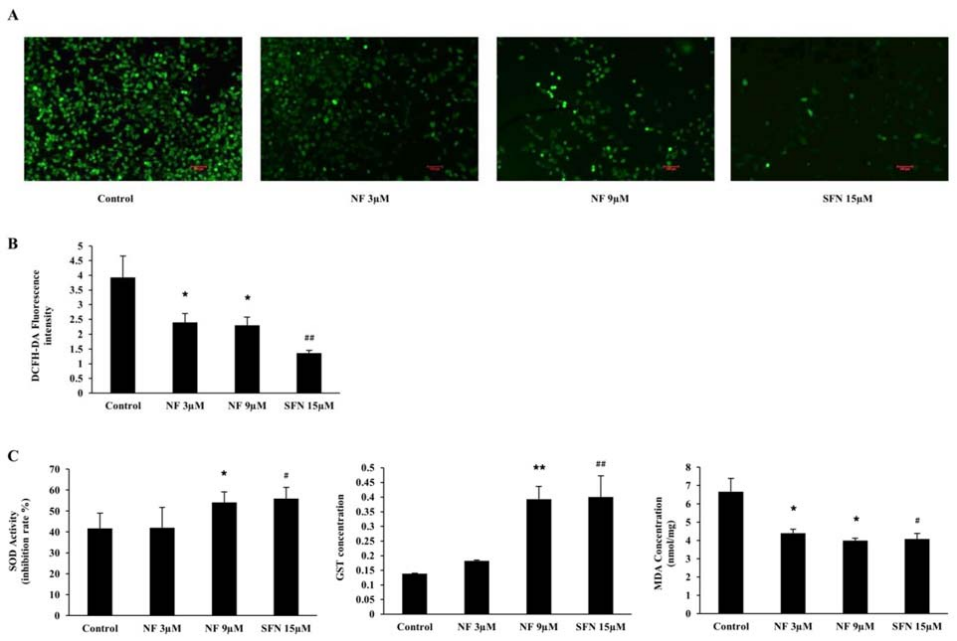
**Protective effect of NF against OS:** Human BPH-1 cells were treated with NF 3, 9 µM and SFN 15 µM for 48 h**. A.** ROS was determined with DCFH-DA staining and observed under Laser scanning confocal microscope (x 100) **B.** DCFH-DA fluorescence intensity representation. **C.** Determination of SOD, GST and MDA activity at 48 h. Values are presented as means ± SD (n = 5). For NF: *P< 0.05, **P< 0.01 compared to control group. For SFN: #*P*< 0.05, ## *P*< 0.01 compared to that of the control group (no treatment).

To examine the defensive role of NF through ROS induced OS, we carried out DCFH-DA staining for ROS detection with or without NF and SFN in BPH-1 cells. Figure 3((A), (B) depicted both NF 3 µM and 9 µM were able to diminish ROS 39.05% and 41.55%, where SFN 15 µM reduced by 65.22% than the control. In Figure 3(C), the activity of SOD was increased in all treatment groups, but NF 9 µM and SFN 15 µM increased by 12.42% and 14.27% respectively compared to control. MDA concentration was dropped by 34.04%, 40.19%, and 38.77% by NF 3 µM, 9 µM and SFN 15 µM, respectively. However, NF 3 µM intensified GST concentration by 24.19%, whereas NF 9 µM and SFN up-regulated almost 94% than control at 48 h treatment.

### NF improves BPH condition by reducing cell proliferation

**Figure 4.**
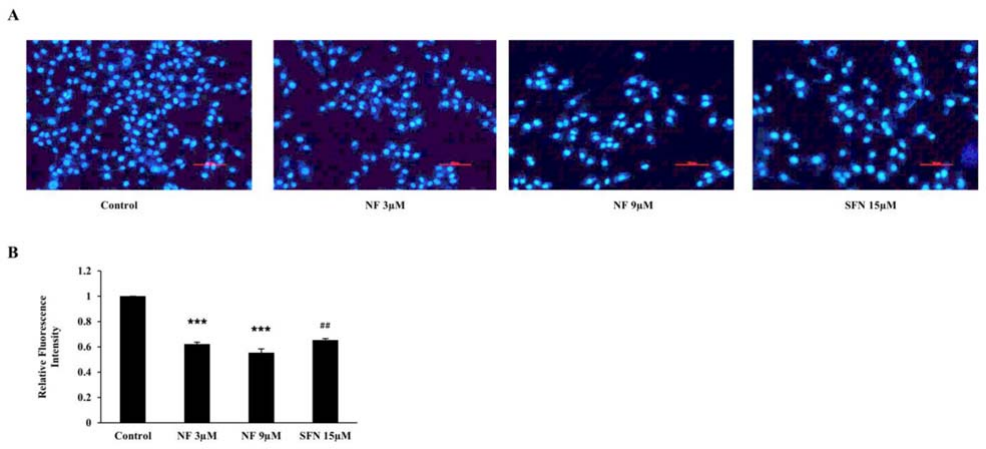
**BPH condition amended by reducing cell proliferation**: Human BPH-1 cells were treated with NF 3, 9 µM and SFN 15 µM for 48 h**. A.** After treated for 48 h, alive cells were determined with immunofluorescence analysis using DAPI staining, **B.** Relative Fluorescence intensity. Values are presented as means ± SD (n = 3). For NF: ***P< 0.001 compared to control group. For SFN: ##*P*< 0.01 compared to control group (no treatment).

To scrutinize the OS state improvement, NF can improve BPH-1 cell proliferation; we treated the cells with or without NF and SFN. After 48 h, cell proliferation was assayed with DAPI staining (Figure 4(A)), and both NF 3 µM and 9 µM could mitigate proliferation by 37.87% and 44.78% respectively nonetheless SFN reduced by 34.60% (Figure 4(B)).

### Modulation of apoptosis by NF

**Figure 5.**
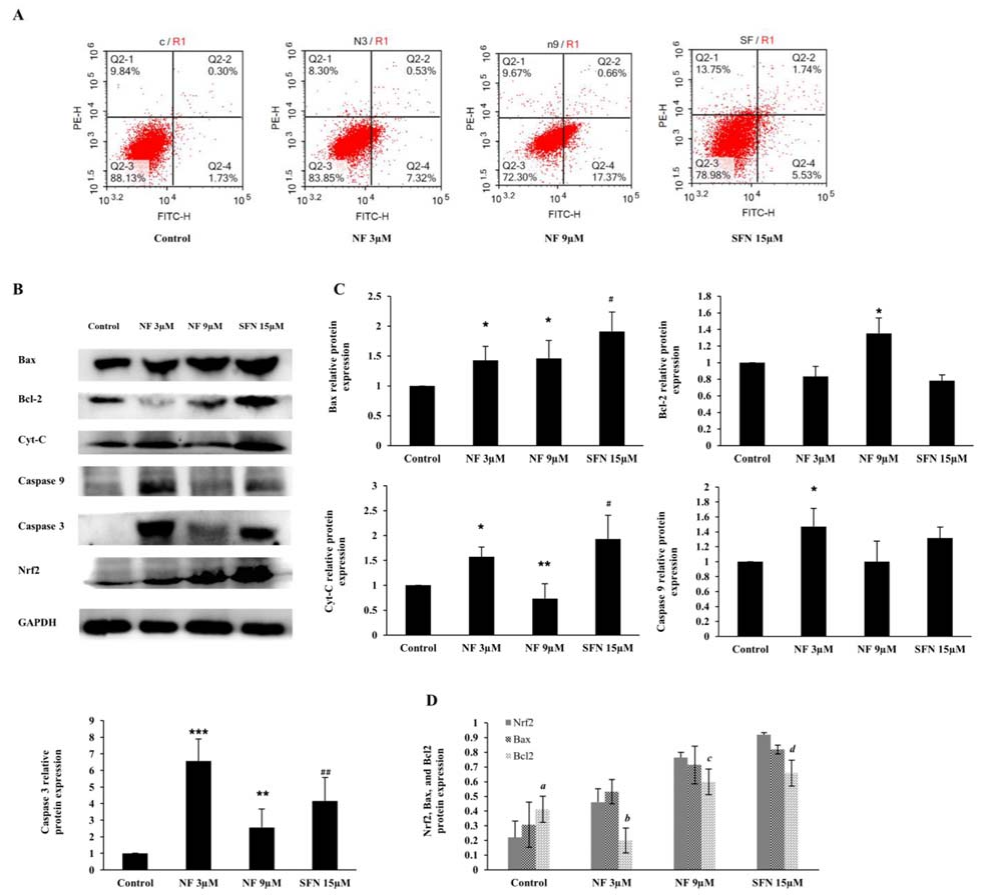
**Apoptotic regulation by NF:** Human BPH-1 cells were treated with NF 3, 9 µM and SFN 15 µM for 48 h**. A.** After treatment, apoptosis was determined with flow cytometry analysis using Annexin V-PI staining, **B.** Protein expression of Bax, Bcl-2, Cyt-c, cleaved caspase 9, cleaved caspase 3 and GAPDH were determined with western blot. **C.** Relative protein expression of Bax, Bcl-2, Cyt-c, cleaved caspase 9, cleaved caspase 3 were normalized with GAPDH. **D.** Comparison of the Nrf2, Bax, and Bcl2 relative protein expression at 48 h and all the values are normalized with GAPDH. Values are presented as means ± SD (n = 3). For NF: *P< 0.05, **P< 0.01, ***P< 0.001 compared to control group. For SFN: #*P*< 0.05, ## *P*< 0.01 compared to control group (no treatment). *a* (*P*< 0.05) compared to the Nrf2 of the control group (no treatment), *b* (*P*< 0.01) compared to the Nrf2 of NF 3 µM group, *c* (*P*< 0.05) compared to the Nrf2 of NF 9 µM group, and d (*P*< 0.05) compared to the Nrf2 of SFN 15 µM group.

The imbalance between cell proliferation and apoptosis is one significant reason for prostate hyperplasia. The most activation of Nrf2 along with OS and cell proliferation alleviation were noticed at 48 h, according to Figures 2–4. Therefore, we investigated the apoptotic regulation of NF at 48 h, and the apoptotic rates were observed by conducting flow cytometry. As in Figure 5(A), the apoptosis in the control group was 2.03%, which was increased by 7.85% and 18.03% with the treatment of NF 3 µM and 9 µM respectively, where SFN 15 µM increased 7.27%. To explore the mechanism of the apoptotic effect of NF, we observed the protein expression of Bax, Bcl-2, Cytochrome C (Cyt-c), cleaved Caspase 9, and cleaved Caspase 3 in Figure 5(B and C) where all of the above proteins except Bcl-2 were increased. Bax and Bcl-2 protein expression were compared to the Nrf2, where interestingly Bcl-2 down regulation was found significant with the NF and SFN treatment (Figure 5(D)).

## Discussion

Benign prostatic hyperplasia is a recurrent cause of lower urinary tract symptoms, particularly in aging men, when it is amplified more than 30 mL of the prostate gland [30]. Testicular androgen enzymes are the key to developing BPH. The enzyme 5α-reductase 2 bound to the nuclear membrane and converts testosterone to DHT, which continues to remain high in aging men [31]. BPH development involves disruption of DHT-mediated homeostasis between cell proliferation and programmed cell death (apoptosis), which allow proliferative processes to predominate [32,33].

Reactive oxygen species (ROS), which is produced by chronic prostatitis, induces prostatic proliferation of stromal and glandular cells and provoke alterations in the DNA repair machinery, point mutations, deletions or rearrangements and contributes to the alteration in the normal regulation apoptosis, thereby leading to the hyperplastic condition [34]. ROS can initiate lipid peroxidation, which can generate peroxyl radicals, alkoxyl radicals, and MDA, which is genotoxic [35]. Nrf2 is a nuclear transcription factor. It acts as a master regulator of the redox homeostasis and is responsible for the expression of HO-1, SOD, NQO1, and GST [36,37]. In the case of BPH condition, Nrf2 was found low in prostate tissue but higher in prostate cancer [38] which indicates that due to the imbalance of the antioxidant tools, oxidative stress heightens in the BPH. In our study, Nrf2 and its downstream HO-1 and NQO1 gene and protein expression were amplified by 3 µM and 9 µM of NF in 24 and 48 h where 48 h treatment illustrated better expression than SFN 15 µM (Figure 2). Under homeostatic conditions, activation of Nrf2 is contrastively regulated by Keap1 by Nrf2-Keap1 complex where Nrf2 need to be dissociated from this complex translocation to the nucleus where it binds to antioxidant responsive element (ARE) and facilitates multiple vital functions including, antioxidant activity, detoxification, maintenance of cellular redox homeostasis [39]. Keap1 is also responsible for Nrf2 degradation in both cytoplasm and nucleus [40] indicates that the less expression of Keap1 leads to more abundance and activation of Nrf2. We found that NF was able to negatively regulate the expression of Keap1 in both gene and protein better than SFN (Figure 2(D, E)) compare to the Nrf2, which may present the mechanism behind the activation of Nrf2 by NF but it needs to explore more to ascertain the precise mechanism. As discussed before, ROS was an elevated stage in the case of BPH [41] and cause lipid peroxidation. The GST gene superfamily defends the DNA from oxidative damage. SOD is a powerful antioxidant in which activity disrupted in BPH conditions [42,43]. In this study, NF was almost equivalent to SFN for diminishing the ROS production (Figure 3(A, B)), alongside escalate the activity of SOD and GST and abate the manifestation of MDA (Figure 3(c)), which contributes to obtainable protection in BPH-1 cells.

**Figure 6.**
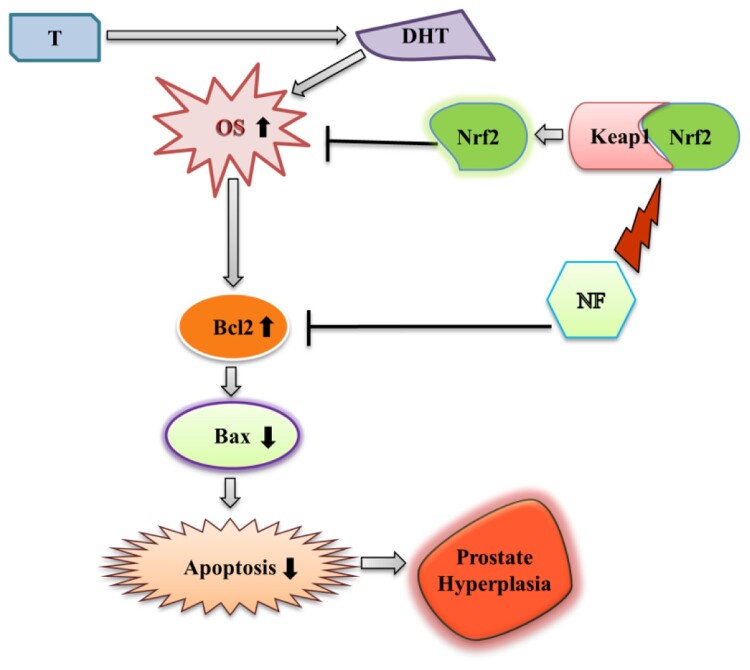
**Modulation of BPH by NF:** This illustrates the possible mechanism of NF to ameliorate BPH condition through regulation of OS and apoptosis. T = Testosterone, DHT = Dihydrotestosterone, OS = Oxidative stress, and NF = Neferine.

In normal healthy prostate cells (WPMY-1), apoptosis induced due to the ROS production [44], but the scenario of BPH is different because of the imbalance between apoptosis and cell proliferation and increased DHT and DHT mediated oxidative stress [45,46]. Excess ROS mediates DNA damage can be sensed by the ATM serine/threonine kinase and Checkpoint kinase protein and activate Tumor protein p53 [47]. Subsequent activation of Bax neutralizes the anti-apoptotic proteins Bcl-2, leading to disruption of mitochondrial outer membrane permeability so that Cyt-c protein typically confined in the intermembrane space spread into the cytosol and activate the mitochondrial-dependent death in the cytosol [48]. Cyt-c binds to the cytosolic Apoptosis protease activating factor-1 and form apoptosome, which recruits initiator pro-caspase-9 allowing to activates downstream executor caspases-3, −6 and −7 for cleavage of cellular substrates leading to apoptotic cell death [49,50]. Nevertheless, in the case of BPH, p53 faces point mutation, followed by the disruption of the normal apoptotic process [51]. Increased ROS production leads to DNA damage, followed by post-translational modification of apoptotic proteins and resist apoptosis [52]. Here, we observed NF was able to reduce cell proliferation (Figure 4) along counterbalancing with OS at the same time (48 h), which gave a hint of apoptotic recovery in BPH-1 cells [53]. In our work, we found NF was superior to stimulate the early apoptosis than SFN (Figure 5(a)), and to explore its mechanism we noticed that Bax, cleaved caspase 9, cleaved caspase 3, Cyt-c protein expression was increased followed by the dropping of Bcl-2 expression (Figure 5(B, C)). Comparison among Nrf2, Bax, and Bcl-2 relative protein expression (Figure 5(D)) might indicate that NF treatment in the BPH-1 cell line triggered Nrf2 activation and regulated Bcl-2 expression the 48 h. In this study, we observed, NF lessened OS by up-regulating Nrf2 meanwhile improved BPH-1 apoptosis, which perhaps suggests a complex interplay between OS and apoptosis. A specific limitation in our present study, including apoptosis regulation by NF or SFN, was either initiated by OS amelioration, or Nrf2 activation was unknown. However, as we discussed, OS related to the proliferation and linked to apoptosis, so NF might present an interaction between OS and apoptosis in BPH-1 cells, which needs further validations (Figure 6).

## Conclusion

This study stipulated NF might protect human BPH-1 cells from OS through ROS inhibition and enhancement of cellular antioxidant defense capabilities by activating Nrf2 and its downstream proteins. NF also regulated the apoptotic protein expression of Bcl-2, Bax, and other related proteins to restore apoptosis and reduced cell proliferation, which possibly indicated the involvement of potential crosstalk mechanisms between apoptosis and OS in the human BPH-1 cells.

## Data Availability

The data have analyzed during the current study are available from the corresponding author on reasonable request.

## Abbreviations

**Abbreviations**: BPH: Benign Prostate hyperplasia; OS: Oxidative Stress; NF: Neferine; SFN: Sulforaphane; ROS: Reactive Oxygen species; MDA: Malondialdehyde.; SOD: Superoxide dismutase; GST: Glutathione-s-transferase; ARE: Antioxidant response element; Nrf2: Nuclear factor erythroid 2-related factor-2; HO-1: Heme oxygenase-1; NQO1: NAD(P)H Quinone Dehydrogenase-1; Keap1: Kelch-like ECH-associated protein-1; Bax: Bcl-2 associated X protein; Bcl2: B cell lymphoma-2; Cyt-C: Cytochrome-C.

## Competing of interest

The author declared that there is no conflict of interest
